# What do we know about the epidermis’ optical properties and their influence on optical device functionality?

**DOI:** 10.1117/1.BIOS.2.3.032505

**Published:** 2025-06-04

**Authors:** Tananant Boonya-ananta, Andres J. Rodriguez, Ajmal Ajmal, JunZhu Pei, Kimberly L. Branan, Amanda Sanchez, Marianne Porras Bouzas, Michael Alzamora, Gerard L. Coté, Jessica C. Ramella-Roman

**Affiliations:** a Florida International University, Department of Biomedical Engineering, Miami, Florida, United States; b Texas A&M University, Department of Biomedical Engineering, College Station, Texas, United States; c Texas A&M Engineering Experiment Station, Center for Remote Health Technologies and Systems, College Station, Texas, United States; d Florida International University, Herbert Wertheim College of Medicine, Miami, Florida, United States

**Keywords:** epidermis, melanin, optical properties, skin tone

## Abstract

We review the published work on the optical properties of the epidermis. Considering three primary skin tone groups, we summarize the existing data on epidermal absorption and scattering coefficient across the 300- to 1000-nm range. We include both experimentally derived values and ones extrapolated through models of bulk skin. We note a paucity of experimental data across all skin tones, which highlights the need for further research on epidermal melanin and skin tone to improve the reliability of optical devices interfacing with the skin.

Statement of Discovery This study highlights the variability in epidermal optical properties. By reviewing absorption and scattering data across various skin tones and modeling light transport with Monte Carlo simulations, it underscores gaps in existing research, particularly regarding the control of demographic factors. The findings call for focused studies on melanin and skin tone to enhance the reliability and inclusivity of skin-interfacing optical systems.

## 1 Introduction

The global wearable medical device market is valued at $33.85 billion in 2023, with a projected compound annual growth rate of 25.7% from 2024 to 2030,^1^ reflecting the soaring popularity of wearable medical technologies. These systems often include optical technologies that utilize light to capture biological health metrics; examples of such systems are photoplethysmographs that measure heart pulse and rate and pulse oximeters that measure blood oxygen levels. Pulse oximetry measurements rose significantly during the COVID-19 pandemic as a critical vital sign determining patient clinical decisions.^2^ Recent studies^3^^,^^4^ have indicated a bias in the blood oxygenation measurements when pulse oximetry devices are used on patients with darker skin tone. These works led the US Food and Drug Administration (FDA) in 2021 to issue a safety communication on the limitations and accuracy of pulse oximeters.^5^ The work of Sjoding et al.^4^ indicates that there is nearly a threefold frequency of occult hypoxemia (over prediction of ${\mathrm{SpO}}_{2}$ from pulse oximeter) in Black patients over White patients that is missed from pulse oximetry measurements. Furthermore, pulse oximeters have provided falsely higher readings for Asian and mixed-ethnicity individuals.^6^ A simulation study^3^ has also demonstrated an overestimation of oxygen saturation measurements from individuals with darker skin tones.

Light propagation is influenced by the body’s optical properties, described here as the absorption and scattering coefficients, but can sometimes include the anisotropy factor. For pulse oximetry, light must propagate through the upper layers of the skin before interacting with arterial blood, where oxygen saturation is measured by calculating the ratio of oxy- and deoxyhemoglobin. Similarly, in photoplethysmography, the assessment of pulse that yields the heart rate and other metrics is influenced by epidermal melanin. Our work^7^^,^^8^ and others^9^^–^^12^ have shown how the signal amplitude is decreased with increased melanin concentration.

For decades, it has been known that melanin in the epidermis acts as a light absorber.^13^ The melanin types and melanosome content determine the pigmentation of the skin, and its absorption coefficient limits the penetration depth of light, with higher magnitudes of absorption at shorter wavelengths, which exponentially decay toward the longer end of the spectrum.

In this work, we conduct a literature review of the epidermis’s optical properties,^14^^–^^20^ specifically the studies that exclusively report the absorption coefficient and reduced scattering coefficient of the epidermis. Our work shows significant variability in the reported values due to biological variables [age, sex, ethnicity, body mass index (BMI), etc.], the different measurement techniques and sample preparation (*in vivo* and *ex vivo*) utilized to obtain optical properties, and the data’s location. The studies generally included a few participants, often not controlling for the biological variables. Furthermore, we highlight studies^7^^,^^8^^,^^16^^,^^21^^–^^25^ that rely on a modeling-based platform to calculate the optical properties of the epidermis. These studies have shown reliance on a variation of a single model based on melanosome volume fraction.^26^^,^^27^ Ultimately, we conclude that there is a lack of a reliable or standardized technique for classifying a melanosome volume fraction for these models based on a stratified skin type scale, such as the Fitzpatrick skin tone scale or the Monk Skin Type scale.^28^ We aim to summarize the published work on characterizing only epidermal absorption and scattering coefficients.

### 1.1 Epidermis and Its Pigmentation

**Fig. 1:**
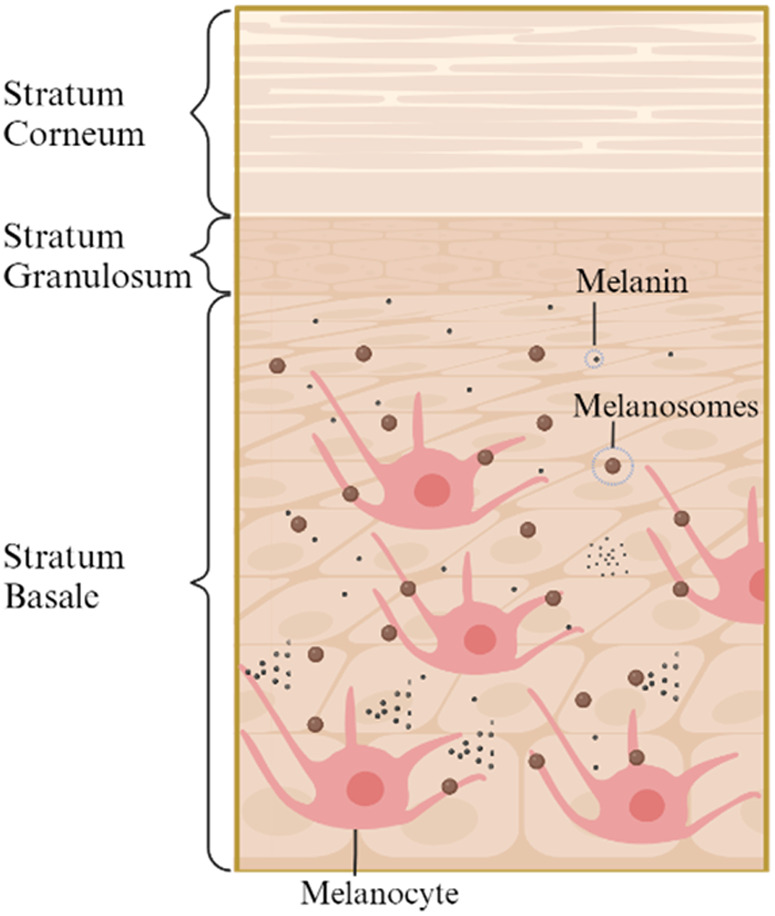
Distribution of melanin and melanosomes in the skin (created with BioRender).^32^

The epidermis comprises five constantly self-renewing layers: stratum corneum (most superficial), stratum lucidum, stratum spinosum, stratum granulosum, and stratum basale (the deepest layer).^29^^–^^31^ Figure 1 is a simplified three-layered diagram of the epidermal layers showing the location and distribution of the main absorbers and scatterers in the deeper layers, namely, melanocytes, melanosomes, and melanin. The stratum basale contains melanocytes and Merkel cells responsible for melanin production and is separated from the dermis by the basal lamina.^33^

In the epidermis, melanin distribution is orchestrated primarily to protect against the harmful effects of ultraviolet (UV) radiation from sunlight.^34^ Studies have shown melanin concentration to be higher in areas of greater photo exposure than in photo-protected skin.^35^ For example, the back of the hand versus the palm can present more than a twofold difference in melanin index.^36^^–^^38^

Melanin diversity and genetic factors create various skin tonalities.^39^ Changes in skin pigmentation result from melanogenic activity, which arises from the size, packaging, and number of melanosomes producing two types of melanin:^40^^–^^42^ eumelanin and pheomelanin.^43^ Eumelanin is responsible for the darker brown–black pigmentation of the skin.^40^^,^^43^ Pheomelanin correlates with pinkish skin color. The combination of the content of these two monomers makes up the total volume fraction content of melanin.^44^^,^^45^ However, this paper focuses on the influence of total melanin and melanosomes on optical properties, not the individual types of melanin.

Total melanin content in the epidermis can differ approximately twofold between Asians and Caucasian individuals and approximately three times between Asians and Black individuals.^40^ This indicates an up to sixfold difference among extreme skin types.^40^ In the range of 650 to 800 nm, the wavelength-dependent optical density of epidermal melanin dictates that individuals with lighter skin possess 1.3% to 6.3% of melanosomes per unit volume in the epidermis.^26^^,^^27^ Moderately pigmented adults have a concentration of 11% to 16%, whereas those with darker pigmentation exhibit a concentration of 18% to 43%.^26^^,^^27^

Clinically, skin tone classification is achieved with various scales. Established in 1975, the Fitzpatrick Scale categorizes skin into six types (skin types I to VI) and was primarily developed based on responses to sun exposure, with different degrees of burning or tanning^46^ and is the most commonly used skin type classification scale compared with von-Lucschan,^47^^,^^48^ Massey–Martin,^49^ and Monk^28^ scales. Despite their utility, these scales lack quantitative precision compared with the usefulness of optical properties when developing wearable devices. Although the connection among ethnicity/race, melanin content, and Fitzpatrick classification is not always direct, throughout this review paper, we will consider that, in general, Caucasian, Mediterranean/Asian, and African/black refer to lightly, medium, and highly pigmented skin, respectively.

## 2 Optical Characterization

Of the many experimental methods used to quantify specifically the epidermis’s optical properties, fiber optics-based techniques,^14^^,^^17^^,^^19^ confocal microscopy,^18^ and spatial frequency domain imaging/spectroscopy (SFDI/S)^15^^,^^50^^–^^52^ are the most common.

**Fig. 2:**
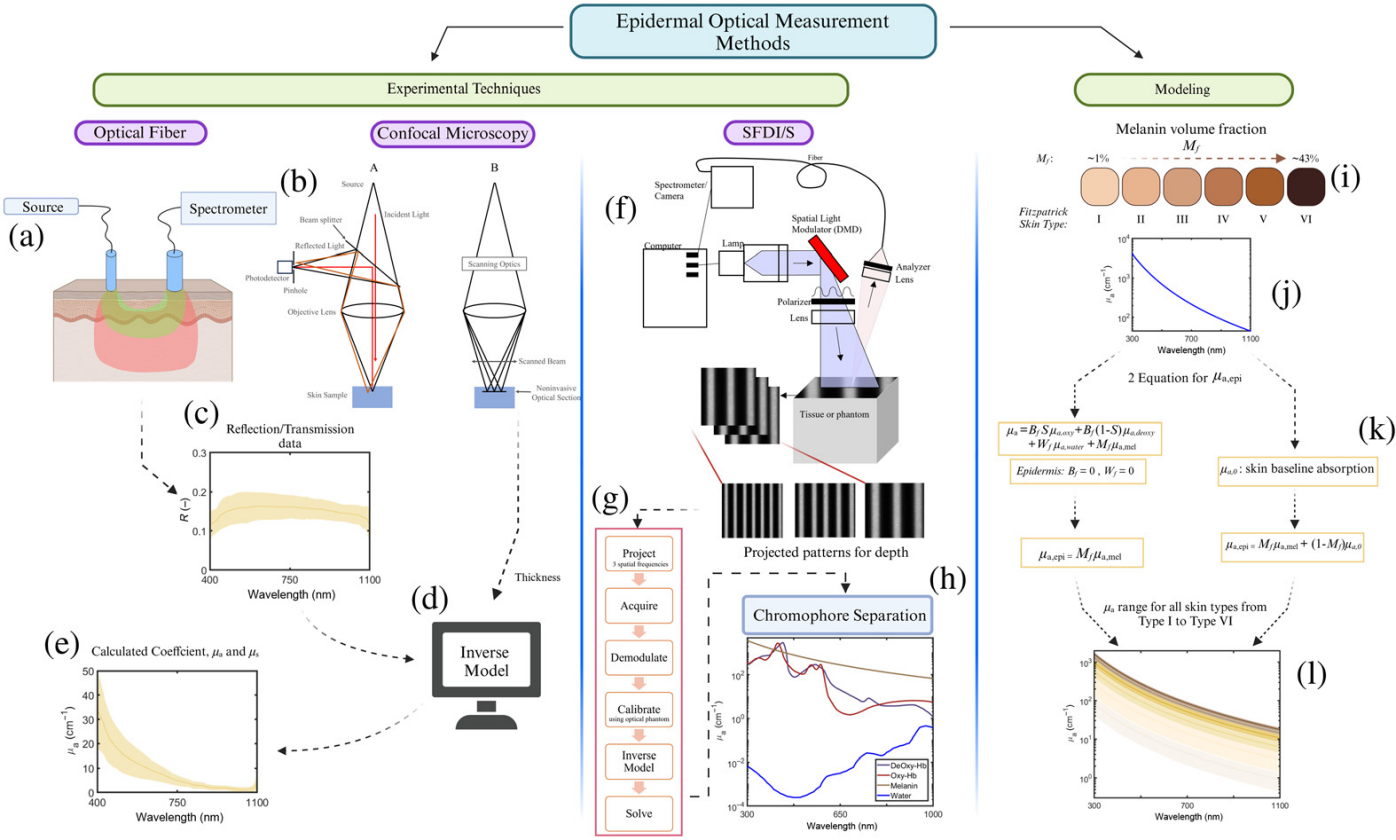
Summary of optical measurement methods that have been used to characterize epidermal properties. (a) Optical fiber measurement technique. (b) Example of a confocal microscopy apparatus setup. (c) From optical fiber, reflectance or transmittance data are obtained over a spectrum. (d) Reflectance and transmission data (and thickness from confocal if available) are input into an inverse model to derive (e) optical absorption and scattering coefficients. (f) SFDI/S example experimental setup with three different spatial frequencies; the image and spectrum are captured and analyzed (g) using a specified method to produce (h) measured optical properties and possible separation of chromophores. (i) To represent the epidermal optical absorption coefficient of a particular skin tone, a melanin volume fraction percentage is selected ranging from 1% to 43% and multiplied by (j) the absorption spectra of melanin (k) to provide a spectral band of absorption coefficient for each estimated skin type (l).

Experimental methods often rely on inverse models to characterize the optical properties; these models are highlighted in the summary Table 1 for each experimental modality. For example, in the case of bifurcated optical fibers with spectrophotometers revealing distinctive spectral patterns^54^ an inverse model based on minimization [Fig. 2(a)] is used. Multidiameter single-fiber reflectance spectroscopy utilizes Monte Carlo simulations to model the skin’s optical properties, quantifying scattering, absorption, and angular intensity variations^55^ [Figs. 2(c)–2(e)]. Optical fiber-based methods, such as diffuse reflectance spectroscopy, analyze skin optical properties across anatomical regions.^23^^,^^54^^–^^59^ SFDI/S [Figs. 2(f)–2(h)] determines skin optical properties by projecting specific spatial frequency patterns onto a surface and capturing the spectrum through a camera or spectrometer.^15^^,^^51^^,^^60^^–^^64^ This technique can be favorable due to its capabilities with image resolution smaller than 1 mm.^62^ SFDI utilizes a Monte Carlo–based model to obtain both bulk and layer properties.^15^ Confocal microscopy with various modalities provides depth-resolved 3D images [Fig. 2(b)], enabling the study of parameters such as melanin content^18^^,^^65^^–^^67^ and depth-resolved sample scanning using a pinhole with the ability to enable the direct visualization of melanosomes.^68^

**Table 1:** Summary of epidermal absorption and reduced scattering across various reported studies.

Instrumentation	Location (author)	Demographic	Wavelength(s) (nm)	Optical property measured					Model used
				$\mu_{a}$ (${\mathrm{cm}}^{-1}$)	$\mu_{s}^{\prime }$ (${\mathrm{cm}}^{-1}$)	Melanin concentration	Others	Comments	Equation-based forward model experiments—inverse model
Models	(Patwardhan, A. P. 2005)	3 pigmentations: light/medium/dark	350 to 700	84/300/680 to 10/30/70	436 to 104^a^	—	g epidermis (0.7 to 0.8) and g stratum corneum (0.9 to 0.94)	Scattering only reported once, not for specific skin type	Equation-based [Eqs. (1)–(3)] calculation of $\mu_{a}$
	(Ajmal 2021)	Fitzpatrick I and VI	523	1.865/127.47	4.94/25.5^a^	3% to 42%	—	Uses MCMatlab-based model	$\mu_{s}$ values vary based on each independent study
	(Rodriguez 2022)	Fitzpatrick I	400 to 1000	21 to 1	40/90 to 23/31	1.3%	—	—	
	(Boonya-ananta, T. 2021)	Fitzpatrick I	660 and 890	0.3442/0.3184	24.2/44.9^a^	3%	—	Uses MCMatlab-based model	
	Face, ventral arm, and dorsal arm (Tsui, S. Y. 2018)	—	410 to 760	117/161/236 to 14.1/19.4/28.5	29/40/136 to 13/18/69^a^	8.23/11.3/17.2%	—	—	
	(Meglinski I.V. 2002/Lister 2012)	Fitzpatrick I	450 to 1050/360 to 740	SC (10 to 1.7)/living Epi (24 to 4.8)/total (6 to 0.6)	90^a^	3%	g=0.8 living epidermis	Assumes wavelength-independent scattering	
	(Svaasand 1995 and Lister 2012)	Light skin	360 to 740	15 to 3.1	80.6 to 39.2	—	—	Indicate blood used in the model (Fig. 3) due to the presence of hemoglobin peak signatures	
	(Jonasson, H. 2018)	Fitzpatrick I	300 to 1000	28/200 to 5.5/0.8	Bulk skin is reported	1.2% to 8.4%	—	$\mu_{s}^{\prime }$ experimental fiber (inverse MC) n=1764 “fair” skin type (bulk)	
	Model (Hu, D. 2019)	N/A	N/A	$0.1\leq \mu_{a}\leq 0.2$	$9\leq \mu_{s}^{\prime }\leq 22$	N/A	—	The first layer’s minimum thickness—for which the first layer’s optical properties could be accurately estimated—could be as small as 0.02 cm	Diffusion approximation of the radiative transfer equation
Fiber	(Marchesini, R. 1992)	10 Caucasians (various locations)	400 to 800	24 to 0.2	32 to 21	—	*Ex vivo*	Thickness 73±24 μm	1D diffusion approximation radiative transfer equation^19^^,^^53^
	(Wan, S. 1981)	Caucasian/dark	250 to 800	650/1050 to 20/100	160/230 to 33/52	—	*Ex vivo*	—	Kubelka–Munk model
	(Shimojo 2020)	15 epidermis, Asian (Fitzpatrick skin type III)	400 to 1100	33 to 1.3	100 to 27		*Ex vivo* (g used 0.8, 0.9, and 0.95)	Thickness 0.09 to 0.32 mm	Inverse Monte Carlo model
	Right forearm (Jonasson, H. 2023)	3526 (“fair” skin)	450 to 850	47 to 3.6	Bulk skin was reported	5% melanin median	*In vivo*	Age range only 50 to 64 years; values are reported as $\mu_{a}*t_{\mathrm{epi}}$	Inverse Monte Carlo model (EPOS)
	(Salomatina, E. 2006)	7 (various locations)	380 to 1600	12.5 to 1.7	107 to 36	—	*Ex vivo*	Confocal was used for thickness and morphology thickness of 60 to 100 μm	Inverse Monte Carlo model (based on an inverse quasi-Newton algorithm and forward Monte Carlo)^18^
SFDI/S	(Weber, J. R. 2009)	1 (Caucasian) (volar forearm)	650	4.5 to 2.5	20 to 17	—	*In vivo*, two-layer fit versus homogeneous fit	Up to 23.5% accurate for $\mu_{a}$ and 3.8% accurate for $\mu_{s}^{\prime }$	Diffusion approximation of the radiative transfer equation
	(Saager, R. B. 2015)	12 subjects (ages 23 to 75) (light to very dark skin types) (dorsal forearm and the upper inner arm)	450 to 1000	140 to 3.8	Bulk skin was reported		*In vivo*, epidermal melanin distribution thickness estimated ∼100 μm	—	Inverse Monte Carlo model

a Original studies reported the scattering coefficient.

When looking at both inverse and forward models^26^^,^^27^^,^^69^^–^^73^ of light propagation in the epidermis and the skin, several assumptions must be considered. For example, the volume fraction of melanosomes in the epithelial layers is often used to obtain the absorption coefficient.^27^^,^^69^^,^^70^ Jacques^27^ proposed a melanosomes volume fraction of 1% to 3% for Caucasian, 11 to 16% for tanned-skin toned, and 18% to 43% for dark-skin toned individuals. The data originate from earlier studies of melanosome absorption^26^^,^^70^^,^^74^ on cutaneous and retinal pigmented epithelia to create a mathematical relationship between melanosome optical properties and wavelength. The melanosome absorption coefficient model was quantified by exposing melanosomes to a nanosecond pulse laser. This absorption coefficient is described in Eq. (1). The distinction between melanosome absorption and melanin must be highlighted as this work specifically observes the interaction with melanosomes. As described by Jacques,^45^ the use of melanosome volume fraction to represent melanin concentration is done because melanin, as a large chain polymer, does not have a specific molecular weight, and histologically, melanosome density is easily obtained and more quantifiable through microscopic analysis of excised tissue. Therefore, to estimate the volume fraction of melanin in a tissue,^45^ the equivalent volume fraction of cutaneous melanosomes ($M_{f}$) is used, multiplied by the melanosome absorption coefficient, yielding the contribution of pigmentation absorption in the tissue.

$$\mu_{a,\mathrm{mel}}(\lambda )=6.6\times 10^{11}\lambda^{-3.33}\;[{\mathrm{cm}}^{-1}],$$ (1)

$$\mu_{a,\mathrm{epi}}=M_{f}\mu_{a,\mathrm{mel}}(\lambda )+(1-M_{f})\mu_{a,0}(\lambda )\;[{\mathrm{cm}}^{-1}],$$ (2)

$$\mu_{a,0}=7.84\times 10^{8}\times \lambda^{-3.255}\;[{\mathrm{cm}}^{-1}].$$ (3)

Saidi^71^ suggested that the absorption coefficient of the epidermis $\left(\mu_{a,\mathrm{epi}}\right)$ is the sum of two main components: the bulk baseline absorption and melanosome concentration.^23^^,^^24^^,^^45^^,^^75^^–^^80^ Melanosome absorption^26^^,^^27^^,^^70^ ($\mu_{a,\mathrm{mel}}$) and concentration ($M_{f}$) are directly correlated to the skin tone and is the primary absorber in the epidermis. The baseline absorption ($\mu_{a,0}$), is the intrinsic absorption of all other substances in the epidermis^24^^,^^69^^,^^71^ except for melanin, which mainly consists of the cellular components of each epidermal layer and contributes to epidermal scattering. Many authors^16^^,^^22^^,^^23^ have simplified the epidermis model to consist only of the melanin (estimated using melanosomes^45^) volume fraction multiplied by the melanosome absorption coefficient. In Table 1, we also report various models^16^^,^^17^^,^^19^^,^^21^^,^^23^^,^^24^^,^^45^^,^^76^ aimed at estimating the epidermal optical properties; the vast majority of these models are based on the work presented in Eqs. (1)–(3).

In the studies that experimentally capture reflectance or transmission data from either *in vivo* or *ex vivo* data, they have used various forms of inverse models to extract the absorption and reduced scattering coefficients of the epidermis. Examples of these models include an inverse Monte Carlo model,^14^^–^^16^^,^^18^ a diffusion approximation^19^^,^^64^^,^^81^ and an older Kubelka–Munk model.^17^ An example of the inverse Monte Carlo model^16^ used to calculate epidermal absorption and reduced scattering coefficient is presented by Fredriksson et al.^82^^,^^83^

## 3 Summary of Optical Properties

Table 1 summarizes the works that have characterized and reported the human epidermis’s optical absorption and reduced scattering coefficients. This table groups each study according to the methods that the authors have used to obtain the optical properties. These methods are separated by model equations, optical fiber measurements, and SFDI/S. Under the modeling group, various authors^7^^,^^8^^,^^16^^,^^21^^–^^25^ have provided their range of epidermal optical property values with the target skin type or melanin concentration. These studies have used variations of Eqs. (1)–(3) to represent the absorption and reduced scattering coefficient across their respective wavelength range. It must be noted that studies that do not directly report epidermal absorption and reduced scattering coefficient values or where the coefficients cannot be calculated are not included in Table 1. Many more studies describe the optical properties of bulk skin,^35^^,^^46^^,^^51^^,^^52^^,^^63^^,^^84^^,^^85^ but we have not included them in this review.

For the studies based on optical fiber measurement techniques,^14^^,^^17^^,^^19^^,^^20^ Wan et al.^17^ and Jonasson et al.^20^ have reported values for Caucasian skin type; Wan et al.^17^ also reported dark skin type, and Shimojo et al.^14^ reported Asian skin type (Fitzpatrick skin type III). Marchesini et al.,^19^ Wan et al.,^17^ and Shimojo et al.^14^ have performed their study on *ex vivo* excised tissue samples using an integrating sphere with 10 samples and 15 samples for Marchesini et al.^19^ and Shimojo et al.,^14^ respectively. Only Marchesini et al.^19^ and Shimojo et al.^14^ have reported epidermal sample thickness. Jonasson et al.^20^ have provided the largest sample population set using *in vivo* sample measurements with a diffuse reflectance fiber probe at the right forearm of over 3500 subjects. However, only the absorption coefficient of the epidermis was reported, but the reduced scattering coefficient was reported by Jonasson et al.^16^ in a previous study on a smaller population in a similar cohort in 2018 using the same measurement system. Marchesini et al.^19^ utilized a one-dimensional (1D) diffusion approximation, Wan et al.^17^ used the Kubelka–Munk model, and Shimojo et al.,^14^ and Jonasson et al.^20^ used an inverse Monte Carlo model to calculate the optical properties. Salomatina et al.^18^ measured seven *ex vivo* samples taken from various locations with a Caucasian skin type sample population. Samples were measured in reflectance mode with confocal microscopy for isolating the epidermal layer, and optical properties were determined using an integrating sphere. Sample thickness was measured using a high-precision digital micrometer. Absorption and reduced scattering coefficients were calculated using an inverse Monte Carlo model from measured reflectance/transmission data. Saager et al.^15^ performed *in vivo* measurements using SFDI at the dorsal forearm and inner arm on a population of 12 but have only provided absorption and reduced scattering coefficients for Fitzpatrick skin type III. An inverse Monte Carlo model was used to calculate optical coefficients.

## 4 Results

**Fig. 3:**
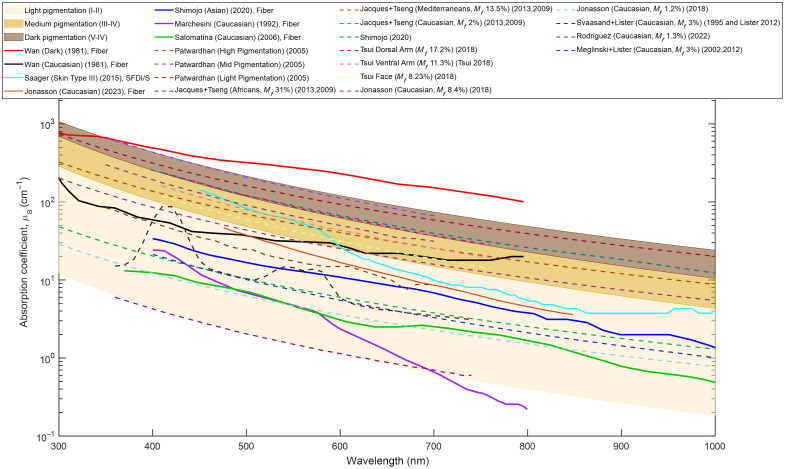
Epidermal absorption coefficient variation across the UV, visible, and NIR spectral range (300 to 1000 nm). Dashed lines are model-derived data, whereas solid lines are experimental values. The legend indicates the authors who have published the data, the skin type classification indicated in their work (melanin volume fraction, $M_{f}$, if available), year of publication, and method. The data have been grouped according to experiment versus model, author, and descending values in which their data appear in the figure. Three color bands in the background represent the spread of the calculated [Eqs. (1)–(3)] absorption coefficient values for three different pigmentation spectra. Light beige (Fitzpatrick skin types I and II), light brown (Fitzpatrick skin types II to IV), and brown (Fitzpatrick skin types V and VI).

Figure 3 includes three background pigmentation bands (light, medium, and dark) for categories of absorption coefficients based on Jacques’^27^^,^^45^ characterization of epidermal absorption coefficient (dotted lines). These three pigmentation bands make up Fitzpatrick skin types I and II (including Caucasian skin), III and IV (including Asian and Mediterranean skin), and V and VI (including African skin type).

This review defines a model as an equation-based mathematical calculation of absorption or reduced scattering coefficient over a specified wavelength range based on assumed volume fraction values and optical properties from other literature. These equation-derived absorption and scattering coefficients are then used in various forward models such as Monte Carlo simulation. Models are based on Eqs. (1)–(3), which generate a continuous range of values across the input spectrum range. Experimental values include measuring the reflectance or transmission of light using various techniques and inverse models to derive the optical properties from collected data.

When comparing the experimental data to the skin tone bands based on equation-based data, it can be seen that the study by Wan et al.^17^ is the only available report of dark skin type and does not have the same trend nor does it remain within the Fitzpatrick skin type V/VI band entirely. In the two studies that report Asian skin type or Fitzpatrick skin type III, the measured values for Saager et al.^15^ start in the skin type III/IV band but dip into the skin type I/II band past 600 nm, whereas absorption for Shimojo et al.^14^ remains within the skin type I/II band. These two studies have different measurement techniques, where one uses SFDI *in vivo* forearm measurements^15^ and the other *ex vivo* sample data from various locations.^14^ The nature of *ex vivo* sample preparation or sample location could be a strong influence on the measured values’ discrepancies. Looking at the four studies^17^^–^^20^ on Caucasian skin type, both Jonasson et al.^20^ and Salomatina et al.^18^ reported datasets that remain within the skin type I/II band, with Jonasson et al.’s study^20^ reporting values almost threefold higher than Salomatina et al.’s study^18^ across the full reported spectrum. The two older studies^17^^,^^19^ report values starting within the skin type I/II; however, Marchesini et al.’s study^19^ showed results that deviate downward under the band at 700 nm and beyond. Wan et al.’s study^17^ reported results that extended upward into the skin type III/IV band at 600 nm and beyond. At the maximum range of 800 nm, the absorption coefficients of these studies differed by two orders of magnitude (100-fold difference). It should be noted that the characterization of the absorption coefficients by the various authors does not extend across the same wavelength spectrum. All authors cover the visible spectrum from 400 to 800 nm. However, Wan et al.^17^ provided results starting at 250 nm and stopping at 800 nm, whereas Shimojo et al.,^14^ Salomatina et al.,^18^ and Saager et al.^15^ started at 400 nm and extended past 1000 nm into the near-infrared range.

**Fig. 4:**
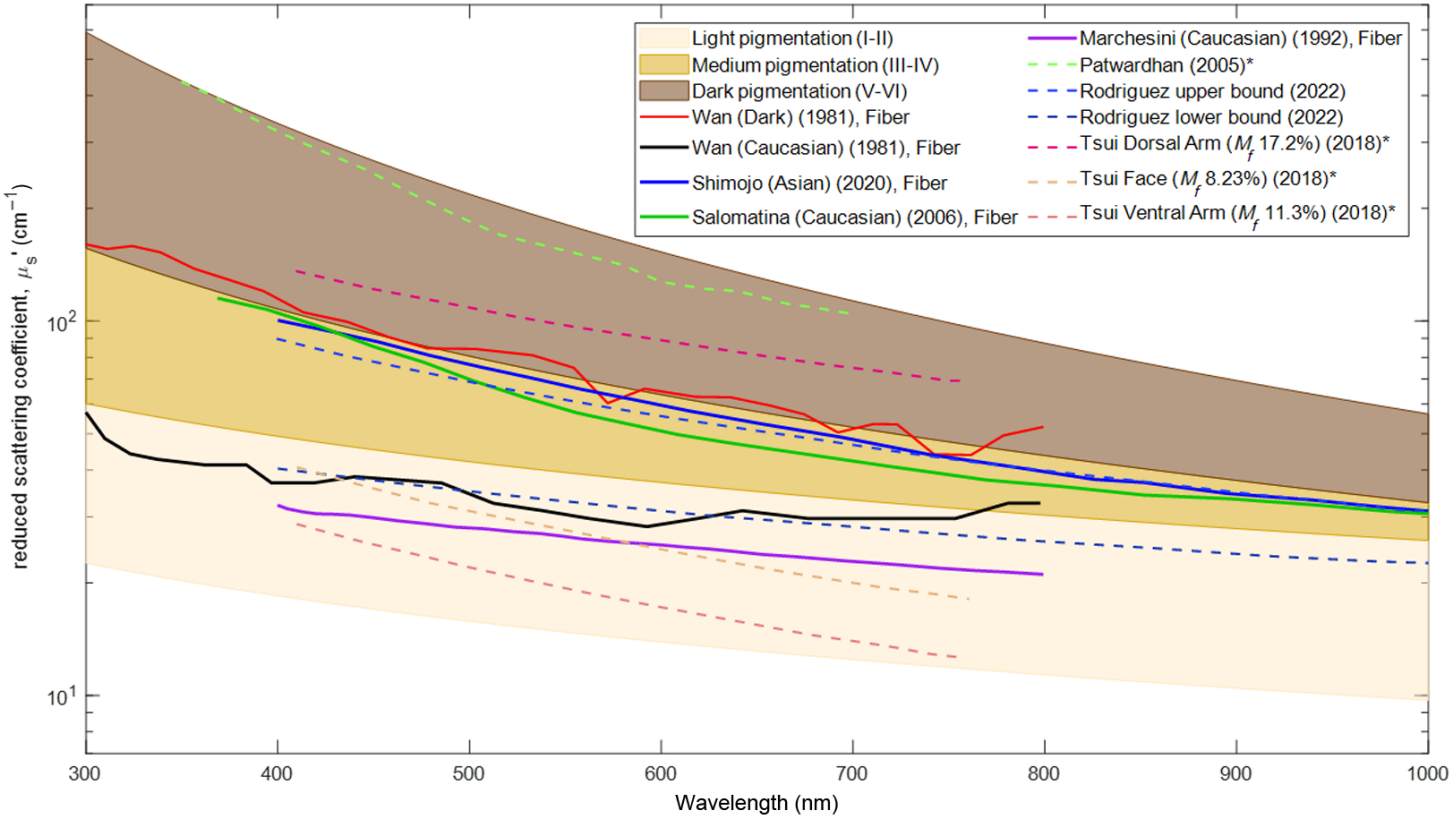
Experimental and model-derived epidermal reduced scattering coefficient values with variation reported across a wavelength range of 300 to 1000 nm. The legend indicates the authors who have published the data, the skin type classification indicated in their work (melanin volume fraction, $M_{f}$, if available), year of publication, and method. The data have been grouped according to experiment versus model (solid versus dashed lines), author, and descending values in which their data appear in the figure. For studies that have reported scattering coefficients (indicated as * in the legend and Table 1) but not anisotropy (g), an anisotropy value of g=0.8 has been used to calculate the reduced scattering coefficient.

In Fig. 4, we observe the reduced scattering coefficient utilizing a similar approach as Fig. 3. The stratification of reduced scattering is done to separate the data available on quantification of the purely epidermal layer. Colored bands are created based purely on available data, which is also used in the plot. For example, the “dark pigmentation” band was created to encompass the highest and lowest values reported. These were classified as “dark pigmentation” by the original authors. This was done to capture all available range including both models and experimental results. The single study of *ex vivo* measurement for dark skin type by Wan et al.^17^ lies on the border of skin types III/IV and V/VI following a general trend dipping back and forth between the two bands. Shimojo et al.’s study^14^ reported values for reduced scattering coefficient that lie within the upper range of the skin type III/IV band following the same general trend. For the Caucasian skin type data, it is apparent that experimental values reported by Salomatina et al.^18^ lie right under Shimojo et al.’s^14^ Asian skin type values, and this similarity results in less than a 15% difference at a wavelength of 600 nm and a 0% difference at a wavelength of ∼430  nm. As for Caucasian skin type values for Wan et al.,^17^ the reduced scattering coefficient lies on the border of skin type I/II and III/IV band. Reduced scattering coefficient values for Marchesini et al.^19^ lie at the top of the skin type I/II color band and showed a similar decreasing tread. The three studies that report Caucasian skin type reduced scattering values were all performed on *ex vivo* tissue samples from different locations on the body.

### 4.1 Impact of Isolated Epidermal Optical Properties on a Common Wearable

We have previously investigated the influence of obesity and skin tone on the functionality of some wearables.^7^^,^^8^^,^^22^ In this review, we extend that work to a range of specific absorption properties representative of the six skin tones, as shown in Figs. 3 and 4. Specifically, we utilized a Monte Carlo program (MCMatlab)^86^ at one wavelength (532 nm) to simulate the alternating component (AC) and baseline component (DC) response of a photoplethysmography (PPG) signal of one watch type (Apple S5) as a function of changes to just melanin content corresponding to the six different skin tones, holding everything else constant. As in the original study,^8^ an Monte Carlo (MC) simulation was used to represent the skin’s optical properties with various levels of skin tone. The MC simulations incorporated a 1.5  cm×1.5  cm×0.3  cm frame at 500×300×3000 elements in each direction. Simulations were carried out using the source detector specifications in Apple S5 (shortest SD separation = 3.3 mm). To account for the lower photon yield at the detectors caused by increased absorption coefficient, simulations were carried out with 100 billion photons. We considered four distinct layers: the epidermis, upper dermis, lower dermis, and subcutaneous fat tissue layers. The pulsatile signature is generated through geometrical changes in blood volume. The associated optical properties of the multilayer skin geometry are given in Table 2; anisotropy was 0.9 for all layers (default), and the index of refraction for the epidermis was 1.47 and 1.4 for all other layers.

**Table 2:** Optical properties of multilayer skin geometry.

Layer	523 nm^27^^,^^45^^,^^86^	
	$\mu_{a}$ (${\mathrm{cm}}^{-1}$)	$\mu_{s}$ (${\mathrm{cm}}^{-1}$)
Epidermis	2 to 246.86	127.47
Upper dermis	1.94	124.54
Lower dermis	17.88	124.54
Fat	1.0e−04	109.78
Blood	149.8	31.87

The signal quality of the PPG signal is analyzed using the AC-to-DC ratio of the simulated waveform. The PPG waveform’s AC-to-DC signal ratio can be calculated by taking the pulsatile (AC) to non-pulsatile (DC) light absorbance ratio. The AC-to-DC ratio percentage change (k-value) is calculated as shown in Eq. (4) to understand the effect of elevated skin tone on the simulated wearable k-value output.

$$k=|\frac{(\mathrm{AC}/\mathrm{DC}{)}_{\text{darker skin}}-(\mathrm{AC}/\mathrm{DC}{)}_{\text{lighter skin}}}{(\mathrm{AC}/\mathrm{DC}{)}_{\text{lighter skin}}}|$$ (4)

**Fig. 5:**
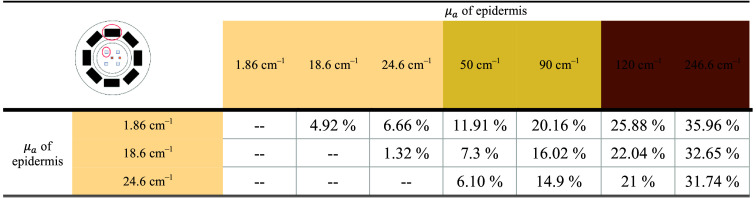
Percentage change in AC-DC ratio (k value) in the skin with increasing epidermal $\mu_{a}$ (${\mathrm{cm}}^{-1}$) values for the shortest S-D separation in Apple S5.

Results in Fig. 5 show the percentage change in AC-to-DC ratio (k-values) between the simulated output of the watch with varying skin tones, indicating signal loss as the epidermis’ absorption coefficient increases. The table offers a comparison of errors across skin tones. This is an example of how results can vary due to epidermal properties based on optical properties in the simulation platform. For example, comparing the signal loss for a fair skin tone level ($\mu_{a}=1.86\;{\mathrm{cm}}^{-1}$) to a dark skin level ($\mu_{a}=246.6\;{\mathrm{cm}}^{-1}$), the signal reduction is 32.65%.

## 5 Discussion

Skin tone variation and its optical quantification are determined primarily by epidermal properties. Quantifying human epidermal optical properties is becoming more critical as different health systems and wearable technologies based on optical methodologies are developed, including in common consumer electronics and medical devices. One proposed method of objective skin pigmentation quantification is the use of a melanometer, which has been reviewed and studied by the FDA.^87^ However, the author suggests several alternative optical approaches to improve accuracy in measuring skin pigmentation for pulse oximetry. The epidermal absorption and scattering properties measured with various instrumentation have been explored here, showing the range and variability in the literature. Specifically, a recently published review by Setchfield et al.,^46^ studied the bulk skin optical properties, looking at the combined effects of skin types on epidermis and dermis absorption and reduced scattering coefficients (not separating specific layers). It highlighted the critical effects of the Fitzpatrick skin type on the optical transmission of light through the skin. Absorption in dark skin is up to 74% greater than in the lower skin pigmentation range, and the variation in each skin type for absorption and reduced scattering can differ by up to 81% and 98%, respectively.

In the experimental epidermal optical characterization literature, not all skin types are represented equally. Of the publications that look at the epidermis, only five performed experimental measurements, and of these studies, only one study from 1981^17^ provided measurements of Black skin type, two on Asian or Fitzpatrick skin types III and IV (one *in vivo* SFDI/S), and three for Caucasian or Fitzpatrick skin types I and II. The largest number of reported *ex vivo* samples that report both epidermal absorption and reduced scattering was 15 samples in Shimojo et al.’s^14^ study on Japanese human skin samples. In the four studies that used *ex vivo* samples of the epidermis,^14^^,^^17^^–^^19^ each followed different methods of epidermal layer separation. In these *ex vivo* techniques, the calculation of the optical properties from reflectance or transmission data relied on an inverse model. The key limitation of these techniques was the requirement of excised tissue samples. SFDI/S^15^ provided an alternative method to measure these properties *in vivo* through a noninvasive technique. This technique can provide a more direct measurement of properties in a specific area, where an optical device is designed to perform its measurements. The work of Saager et al.^15^ demonstrated promising results in separating layered optical properties *in vivo* through the use of SFDS. Jonasson et al.^16^^,^^82^ have shown results specifically of *in vivo* bulk reduced scattering coefficient with a large sample of over 1700 individuals (both men and women) classified as “fair” skin type between the ages of 50 and 64. Their reduced scattering coefficient was calculated through a multilayer inverse Monte Carlo model with data from the EPOS system.^82^ The EPOS system used in the 2023 study,^20^ provided significant progress by expanding the number of subjects to include 3526 individuals and by reporting absorption and reduced scattering coefficient data over a range of BMI as well as including a longitudinal study over 12 months. However, there is a similar lack of representation of dark skin tones in the study, as Fitzpatrick skin type III and greater is not represented. The study is also limited by the age range used, only capturing the span of 14 years (50 to 64 years) on older individuals, but the most significant limitation was in the calculation of the epidermal absorption coefficient where it was assumed that the epidermal thickness was 0.063 mm for all individuals (3526 samples).

Overall, understanding epidermal thickness, which varies across different samples and locations and is not controlled for among experimental studies, is a critical component in using these methods. For example, the SFDS/I system projects different spatial frequencies to probe different depths, and inverse models often rely on a sample thickness input.^20^^,^^82^ Optical coherence tomography (OCT) is a method that can provide direct visualization of the physiological geometry and can be used to resolve properties such as epidermal thickness.^88^ However, OCT is not particularly useful as a stand-alone system for obtaining the optical properties as it provides an overall attenuation coefficient,^88^^,^^89^ which does not separate the absorption from scattering. Thus, our study has touched upon OCT but is not specifically highlighted due to its inability to directly provide absorption or scattering coefficients.

The available experimental^14^^,^^17^^–^^19^ data also suffer from few participants rarely controlling for biological variables such as age, gender, and BMI (only separated by Jonasson et al.^20^ for females). The differentiation across individuals with varying skin tones is often not quantitative, rarely relying on stratified skin type scales such as Fitzpatrick or Monk.^17^^,^^25^ There is no standardization for representing each skin tone in models. The calculation of the epidermal absorption coefficient often relies on selecting a specific melanosome volume fraction ranging from 1% to 43% to represent a range of light, medium, and dark skin tones.^27^ Each of the studies that use a forward model selects a specific melanosome volume fraction estimation based on the skin type of interest. This highlights that, although the volume fraction has been differentiated in various bins (light, medium, and dark), there is no reliable or standardized technique for selecting the appropriate melanosome volume fraction for a target skin type. These volume fractions remain to be mapped to any skin type scale. Mapping to a more stratified scale has a higher probability of creating a more representative match to actual melanin content in the skin. Data from models often refer to the previous work of Jacques and McAuliffe,^26^^,^^27^^,^^45^^,^^76^ citing the epidermal properties as a linear combination of chromophore volume fraction, specifically melanin/melanosomes in the epidermis.^21^^–^^24^^,^^45^^,^^76^ It should be noted that any mathematical model requires significant simplification to characterize a complex biological structure such as the skin. In Figs. 3 and 4, we have created skin type bands trying to capture the range of light, medium, and highly pigmented skin absorption and reduced scattering coefficients. These bands coincided with model data based on melanosome volume fraction values (1% to 43%) estimated for each skin type. However, it should be noted that the two experimental studies that capture medium-pigmented skin (Fitzpatrick skin types III and IV)^14^^,^^15^ do not lie within the band for that skin type band.

Inverse adding doubling (IAD) is one of the most commonly used methods using the integrating sphere to obtain optical properties. However, studies covered in this review lack measurement of the optical properties of human epidermis using the IAD method, likely because MC inversion provides a more flexible approach for handling the complex, multi-layered, and highly scattering nature of human skin. A key challenge with IAD for thin samples such as the epidermis is its assumption of a plane-parallel, homogeneous medium, which can lead to inaccuracies due to increased light losses, boundary effects, and the difficulty of separating scattering from absorption in such thin layers.

In Monte Carlo simulations, the reduced scattering coefficient remains constant and independent of skin type, with variations applied only to the absorption coefficient. In multilayered, voxelized Monte Carlo software, including MCMatlab, scattering varies only with wavelength. This choice was made given that absorption is the dominant optical property influencing light interaction with the skin, and no clear trend exists between skin tone and reduced scattering, as experimental data show scattering values clustering similarly across different skin types. Figure 4 highlights the limited experimental studies on reduced scattering, showing that values cluster similarly across skin types (light/Caucasian, medium/Asian, and dark). Three of the five experimental classifications confirm this clustering, whereas the other two focused on lightly pigmented skin, which skew toward the lower band. This underscores the scarcity of experimental data on epidermal reduced scattering. The clustering of the data is an indication as to why scattering and thus reduced scattering is taken to be constant across different skin types and only varying with wavelength. Further investigation on the measurement of the reduced scattering coefficient and the anisotropy factor would provide greater insight and be a better guide for models and phantoms to correctly represent varying skin tones.

Although it is true that in the Monte Carlo simulations, the calculated reduced scattering coefficient lies at the minimum of the bands at 523 nm, as indicated in Fig. 4, these simulations focused on the impact of the change to absorption coefficient due to increased levels of melanin concentration with increasing skin tone. As many simulations in the literature keep their scattering coefficients and anisotropy values constant, and the lack of experimental data for the scattering coefficient of the epidermis as it relates to different levels of skin tone, we have used the default values found in MCMatlab and kept it constant as well. Of the studies that do report anisotropy, values range between 0.7 and 0.9. With these simulations, we have touched the surface of adjusting epidermal optical properties due to skin tone variation; further investigation could shed light on the impact of change in both scattering and anisotropy of the epidermis as it relates to skin tone across the population.

## 6 Conclusion

The current knowhow on optical properties of the epidermis has been summarized in this paper. Over the years, this insight has been very valuable when designing optical systems interfacing with the skin; nevertheless, low sample size, limited ethnic diversity of the populations monitored, difficulties with sample preparation, and instrument limitations have contributed to the paucity of experimental data. Improved experimental characterization, considering factors such as skin tone (e.g., using devices such as a colorimeter^35^ or melanin content measurement unit), thickness, and other population demographics (gender, BMI, etc.), is essential for improving this field. Embracing techniques such as SFDI/S for *in vivo* measurements or non-linear microscopy presents a pathway toward more reliable data collection. However, overcoming challenges related to accurately separating epidermal layers and controlling tissue variations requires standardizing instrumentation, methodologies, and models with which we can work to support the development of optical methodologies, ensuring their effectiveness in both clinical applications and consumer electronics.

## Data Availability

The tabulated data for all figures and tables are publicly available at https://github.com/FIU-MPL/EpiData.git. The code to generate all resultant figures is included and is created using MATLAB.
